# Harnessing the synergy of statistics and deep learning for BCI competition 4 dataset 4: a novel approach

**DOI:** 10.1186/s40708-025-00250-5

**Published:** 2025-02-15

**Authors:** Gauttam Jangir, Nisheeth Joshi, Gaurav Purohit

**Affiliations:** 1 Banasthali Vidyapith, Tonk, India; 2 Central Electronics Engineering Research Institute, Pilāni, India

**Keywords:** BCI (Brain Computer Interface), EEG (electroencephalogram), Electrocorticography (ECoG), Event Related Potential (ERP), Motor Imagery (MI), Visual Evoked Potential (VEP)

## Abstract

Human brain signal processing and finger’s movement coordination is a complex mechanism. In this mechanism finger’s movement is mostly performed for every day’s task. It is well known that to capture such movement EEG or ECoG signals are used. In this order to find the patterns from these signals is important. The BCI competition 4 dataset 4 is one such standard dataset of ECoG signals for individual finger movement provided by University of Washington, USA. In this work, this dataset is, statistically analyzed to understand the nature of data and outliers in it. Effectiveness of pre-processing algorithm is then visualized. The cleaned dataset has dual polarity and gaussian distribution nature which makes Tanh activation function suitable for the neural network BC4D4 model. BC4D4 uses Convolutional neural network for feature extraction, dense neural network for pattern identification and incorporating dropout & regularization making the proposed model more resilient. Our model outperforms the state of the art work on the dataset 4 achieving 0.85 correlation value that is 1.85X (Winner of BCI competition 4, 2012) & 1.25X (Finger Flex model, 2022).

## Introduction

THe brain is the most active organ of the human body that takes input, processes them, and gives output. Fingers play an important role in human life that is why one of the active fields for rehabilitation is the Brain-Computer Interface (BCI) where fingers and EEG signals are studied together for the normal routine return of a physically challenged or locomotive disabled person. The BCI is connecting path between the brain's electrical signals and external devices like a robotic hand. Hens Berger’s requirements gave birth to EEG, and from that point, onwards many researchers started working in the direction of BCI [1]. The brain generates patterns according to actions as stated in the BCI experimental work by Vidal [2].

Donoghue demonstrated on a monkey that using cortex signal, the mouse pointer can be controlled, even-though accuracy is not up to the acceptance level at that time [3]. Kim et al. predicted that in the future, body parts' actions can be controlled by the thoughts of the brain and it will be really helpful for people who are paralyzed with nerve or limb injuries [4]. They experimented on a monkey, that is sitting on special chair and a lever is there. Two light bulbs are there in the position of the left and right directions in front of the monkey. Jahankhani et al. worked on the EEG signal classification [5]. They used wavelet features and further neural networks. Kubanek et al. worked in this direction to decode the flexion of an individual finger from EEG signals [6]. Liang et al. also worked on an individual finger movement dataset (BCI competition 4 Dataset 4) and Flamary et al. also worked on the same dataset for competition. Liang et al. used a linear regression model and achieved a 0.48 correlation value. Blankertz et al. demonstrated the experiment related to the information transfer rate on six healthy subjects [7]. In the same direction, Santhanam et al. designed a system, in which EEG electrodes were implanted in the monkey dorsal premotor cortex [8]. They achieved a higher information transfer rate by selecting an accurate key selection system. Viola et al. worked on the identification of independent components from EEG artifacts for that 11 user’s data was used [9]. Nolan et al. has done advancement by developing an automatic threshold tool for the removal of artifacts from EEG data [10]. Shou et al. detected EEG spatial-spectral temporal signature error by using the ICA and found that independent component analysis is the best for the specified purpose [11]. Slobounov et al. identified the activity that was induced by visual perturbation [12]. Chaudhary et al. did progress in a brain-computer interface for communication and rehabilitation [13]. Their outcome suggested that non-invasive based BCI along with physiotherapy is an economically promising option for locked-in syndrome patients. Oliveira et al. used a mobile phantom head device for the brain signals intending to reduce the EEG artifacts [14]. Davis et al. analyzed the effects of video quality on EEG signals [15].

Shantala et al. presented the control of a robotic arm by using brain signals [16]. Rosca et al. analyzed the principle to control the robotic arm for the replacement of a human arm just by using the brain's electrical activity [17]. Schwemmer et al. stated that a deep neural network can be used to extract the pattern of index and wrist movements of the subject [18]. Jiang et al. demonstrated the collaboration between brains [19]. They developed brain-to-brain communication by using the electroencephalogram and transcranial magnetic stimulation. Larico et al. developed a system to evaluate attention based on gender and age group [20]. Zhang et al. described a new model to differentiate between emotion and task by using brain signals [21]. Krishna et al. demonstrated the recognition of silent speech by using EEG signals [22]. Kumar et al. proposed a convolutional neural network for the movement intention of rehabilitation robots [23]. Ma et al. proposed a new algorithm for classification and reduced time complexity [24]. They stated that the algorithm has reduced individual differences. Yusoff et al. analyzed the proposed reactions to arousal-evoking stimuli [25]. Cai et al. constructed a model for feature-level fusion of EEG of subjects [26]. Kumar et al. proposed a real-time computational model for cognitive engagement assessment for rehabilitation robots [27]. Lou et al. developed a brain-computer interface system for the betterment of life for people who are suffering from motor disabilities [28]. Zapala et al. studied the effects of handedness on sensorimotor rhythm and BCI control [29]. Dimitrov et al. proposed a system with a better classification of brain signals [30]. Gao et al. proposed a new deep learning framework for better classification on EEG data for emotions recognition named as a channel-fused dense convolutional neural network [31]. Issa et al. presented a new approach for classification of the emotions on the public dataset SEAD and MAHNOB-HCI [32]. Chen et al. presented a hybrid brain-computer interface for the classification of motor imaginary task to control the quadcopter [33]. Roy et al. analyzed EEG signals to control the home appliances [34]. They used the long-short term memory algorithm for the temporal classification and random forest classifier for the classification purpose.

Wang et al. proposed a new way for the recognition of emotion by using deep convolutional neural network and EEG-based electrode-frequency distribution map with a short-time Fourier transform [35]. Amin et al. presented a multilevel convolutional neural network for the extraction of dynamic correlation from EEG motor imaginary data [36]. Ngo et al. worked to control the wheelchair using Neurosky Mindwave headset [37]. Devasia et al. also implemented it on the wheelchair [38]. Little et al. used machine learning to predict elbow motion control [39]. Nasir et al. worked to control the rover for domestic applications by using EEG signals [40]. Alzahrani et al. differentiated between the conventional and tri-polar electrodes for better stability [41]. Mihelj et al. processed neural signals to identify mental individuals [42]. Liu et al. extended the concept of a virtual sixth finger to control [43]. Wenhao et al. used a neural network to classify the fingers [44]. Lee et al. used a deep neural network to classify the finger from ultra-high-definition neural signals [45]. Wang et al. classified the hand motor imagery task [46]. Shahini et al. identified the hand movement intention automatically by using the convolutional neural network [47]. Illman et al. identified the rolandic beta rhythm from MEG and EEG signals from statistical analysis [48]. Mwata-Velu et al. used empirical mode to identify the individual finger movement with the help of LSTM network [49]. Liang et al. developed the system for the training and rehabilitation of finger by using the neural signals to control [50]. Ishibashi et al. stated the work regarding the asymmetry in interhemispheric as movement was performed for left and right hand [51]. Yao et al. used riemannian features for fast prediction of finger movement and machine learning was used for the support [52]. Zheng et al. given the insights for the rehabilitation of patients by the calculation of correlation value [53]. Azizah et al. identified the channels that are most suitable to predict the finger movement [54]. Aziz et al. worked to control the prosthetic hand by using the EEG signals for the stroke patients [55]. Sharma et al. identified the finger flexion based on the variation decomposition by using the BCI competition IV dataset [56]. Al-qaysi et al. sheds light on efficacy and potential in enhancing cognitive abilities by using the smart training environment. Rajput et al. showcased results in leveraging pre-trained models to enhance diagnostic accuracy for classification of magnetic resonance images related to brain tumor. Kaur et al. presented a promising avenue for precise and efficient medical image analysis in neurology. Swetha et al. highlighted advancements in signal processing techniques to improve the accuracy and reliability of brain-computer interfaces using the steady state visual evoked potential.

This paper, presents a work on the finger movement control using EEG signals. First section covers in-depth statistical analysis to find important insights of data from BCI competition 4 dataset IV. In next section data pre-processing is covered to remove the outliers. The cleaned dataset is visualized to identify appropriate activation function inferring the data patterns. A novel deep learning model is developed named BC4D4 incorporating dropout and regularization. Finally, a comparison of various models including winner models from Dataset 4 competition and current state of art model is presented.

## Statistical analysis

The statistical analysis reflects the nature of the dataset. Descriptive statistics describe the data features and summarize them in tables or graphs. Inferential statistics are used to find the relationship and generalization in the dataset. Statistical analysis also reports the outliers in the dataset. In this work, the statistical analysis is applied on BCI competition 4 dataset iv, outcome is reflected in outliers and noisy channels.

**Fig. 1:**
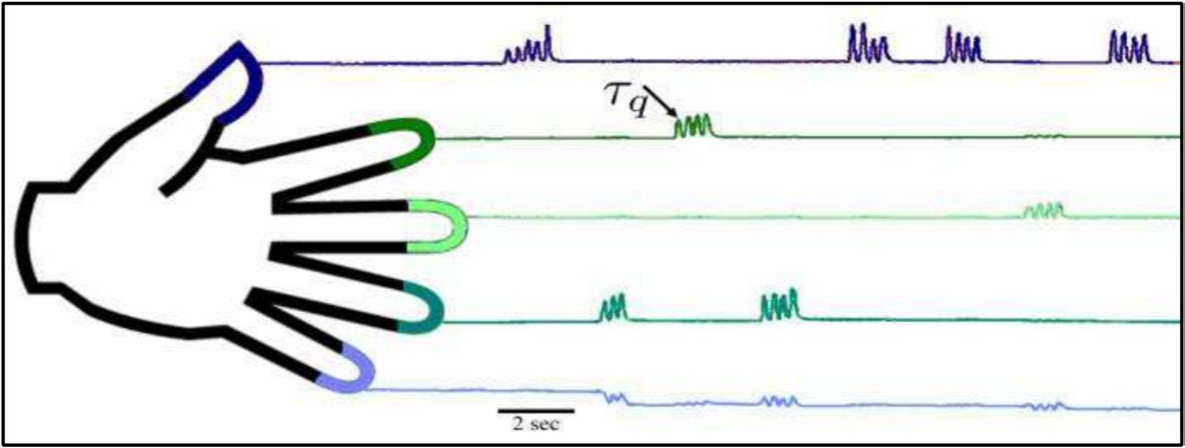
Capturing individual finger flexion [57, 58]

BCI competition 4 dataset iv is the data of three epileptic patients. Subject 1 has 62 features, subject 2 has 48 features and subject 3 has 64 features. A subject (patient) task is to focus on the display and move the respective finger according to finger name or cue (“thumb”, “index”) as displayed on a display device (monitor) as shown in Fig. 1 [57, 58]. Each word is displayed for 2–3 s followed by a 2–3 s rest state. There were near about 30 movements for each finger and the length of each duration was 10 min per subject.

**Fig. 2:**
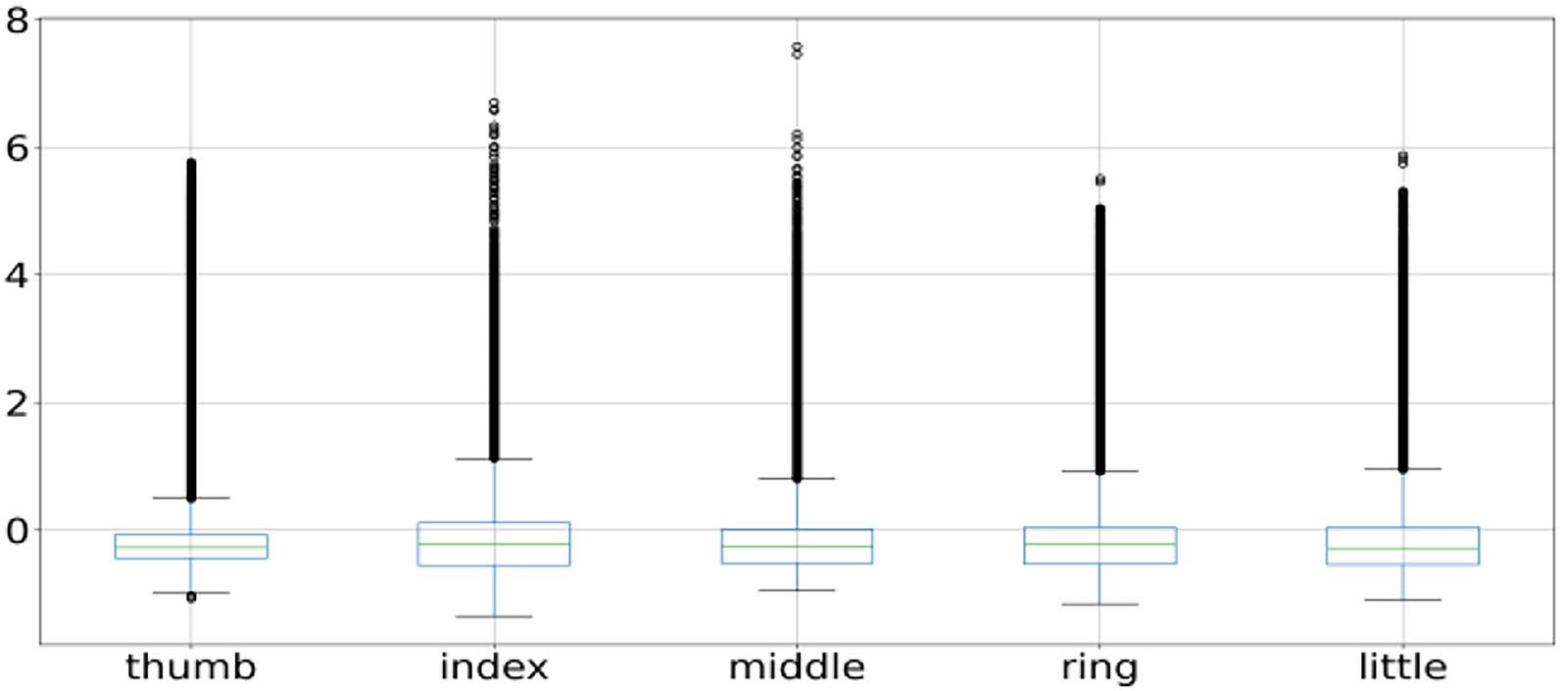
Subject 1 fingers

**Fig. 3:**
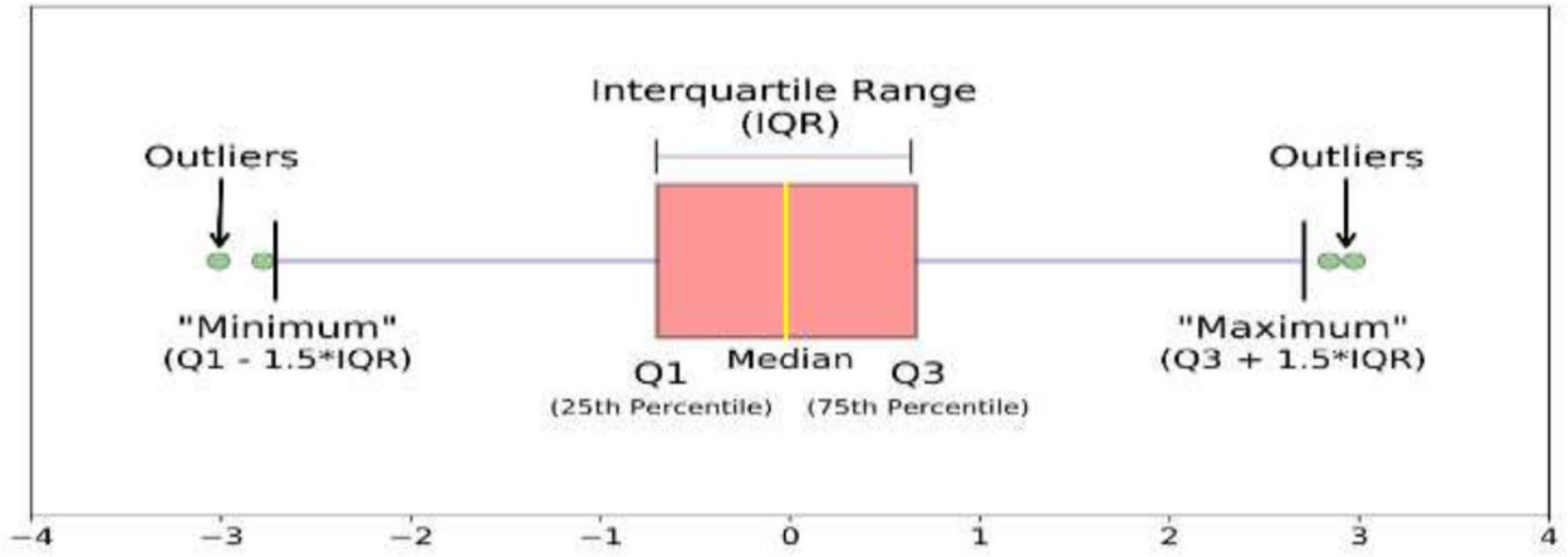
Box Plot (five-point summary)

**Fig. 4:**
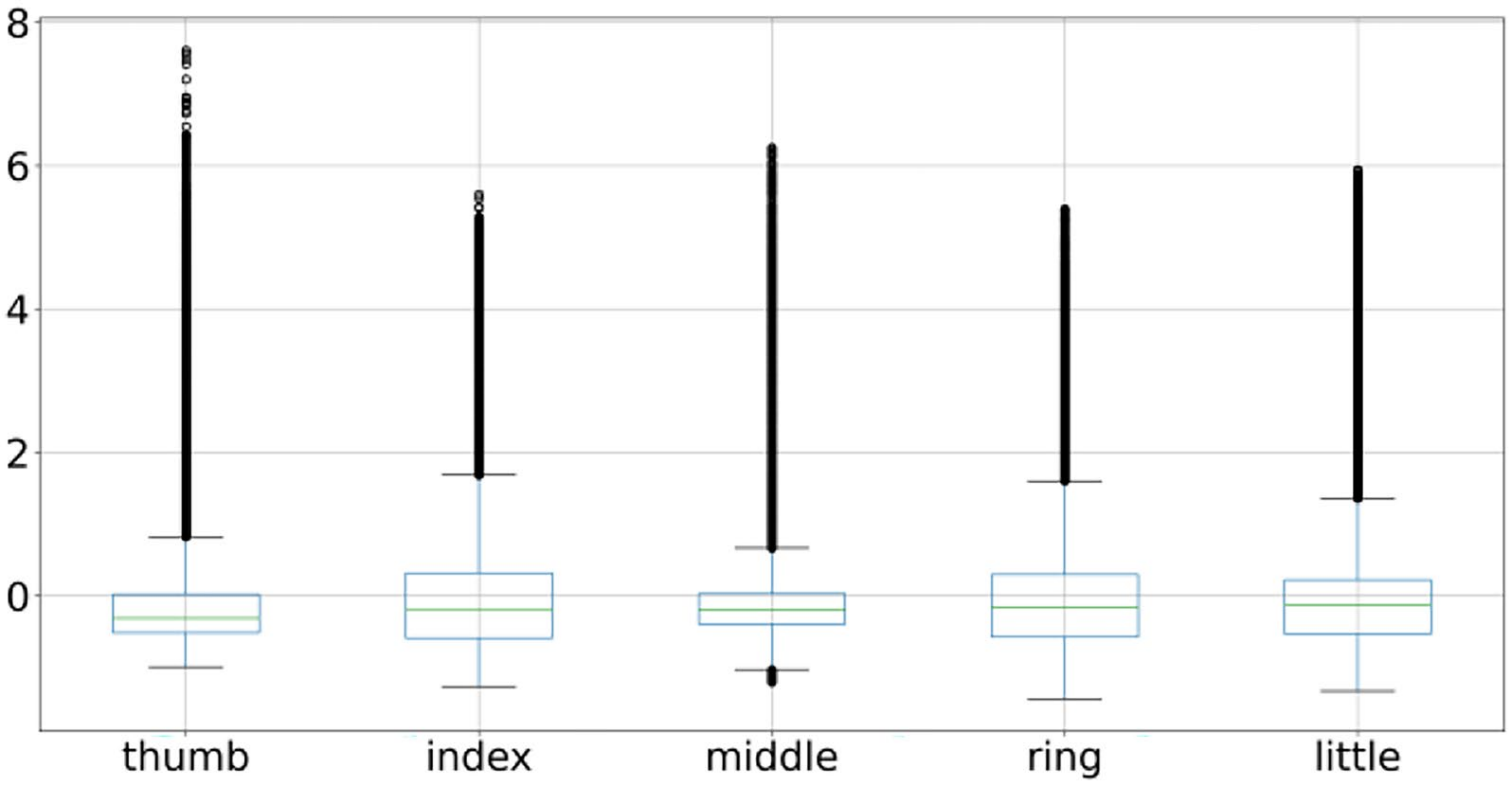
Subject 2 fingers

**Fig. 5:**
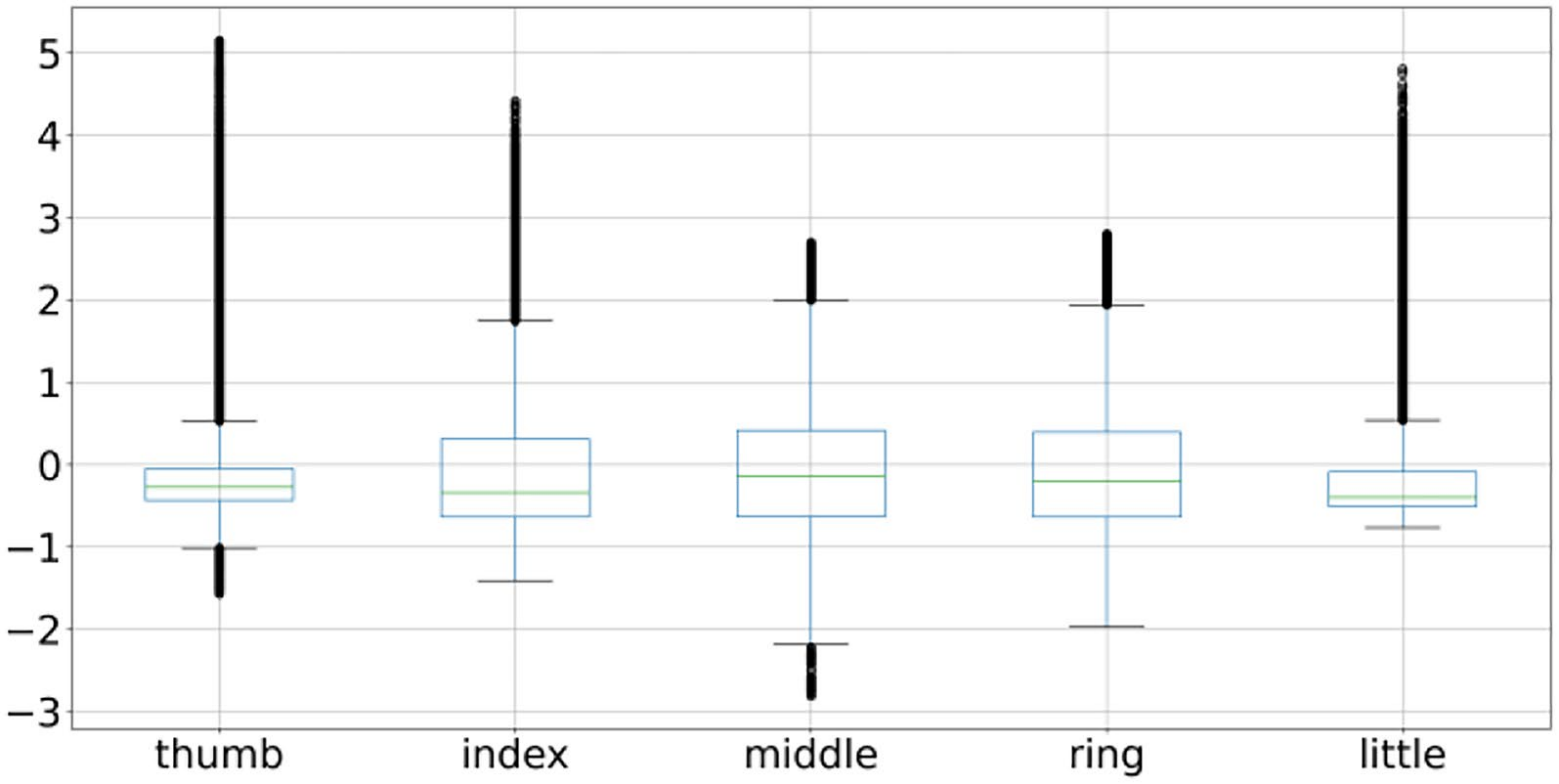
Subject 3 fingers

A box plot is used to plot the overall behavior of the features and the same is applied to all 3 subject fingers movement as shown in Figs. 2, 4, and 5. Box plot summarizes a five-point summary that is minimum, Q1, median, Q3, and maximum as shown in Fig. 3.

A data point that is outside the minimum and maximum is considered an outlier. Two data points as shown in Fig. 3 are outliers as they are outside the minimum and maximum range, so the box plot systematically reveals outliers and identify noise in the dataset. Subject 1 finger movement is shown in Fig. 2 using a box plot. It is a clear reflection from Fig. 2 that outliers are there in all fingers of subject 1. Subject 2 and subject 3 fingers movement is shown in Figs. 4 and 5 by using the box plot and the same kind of noise reflection is also there. Some of the fingers have less noise but most of them have a large amount of noisy data. To get better insights of dataset values like mean, standard deviation (SD), minimum, quartile at 25%, 50%, 75%, and maximum value are shown in Table 1 for subjects 1, 2, and 3 respectively. Descriptive information of subject 1 like its mean value of thumb (−0.0098) and minimum value (−1.1) have a difference of ≈ 1.1, whereas the difference between its maximum & mean values is ≈ 5.8 which is large. Further, its quartile values 25%, 50%, 75% are close to mean whereas max value is beyond quartile range. Hence data distribution is abrupt which indicates noise in it.

**Table 1:** Descriptive information of subject 1,2 & 3

Stats	Finger				
	Thumb	Index	Middle	Ring	Little
Count	60 K	60 K	60 K	60 K	60 K
Subject 1					
Mean	−0.01	−0.01	0.00	−0.01	0.01
Std	1.00	0.99	1.00	0.99	1.00
Min	−1.07	−1.35	−0.95	−1.16	−1.10
25%	−0.44	−0.55	−0.52	−0.53	−0.54
50%	−0.27	−0.22	−0.26	−0.23	−0.28
75%	−0.07	0.12	0.01	0.05	0.05
max	5.76	6.70	7.58	5.51	5.88
Subject 2					
Mean	−0.01	0.01	0.01	0.02	0.02
Std	1	1	1	1.01	1
Min	−1	−1.27	−1.21	−1.44	−1.33
25%	−0.52	−0.59	−0.39	−0.58	−0.54
50%	−0.32	−0.19	−0.2	−0.17	−0.14
75%	0.02	0.32	0.03	0.29	0.22
max	7.61	5.6	6.25	5.39	5.94
Subject 3					
Mean	0.01	0.01	0.01	0.03	0.01
Std	0.99	0.99	1	0.99	1
Min	−1.57	−1.4	−2.81	−1.97	−0.7
25%	−0.43	−0.6	−0.63	−0.64	−0.5
50%	−0.28	−0.3	−0.14	−0.2	−0.4
75%	−0.05	0.31	0.42	0.39	−0.09
max	5.15	4.42	2.7	2.8	4.8

Likewise, other fingers of subject 1 have similar data distribution having outliers in it. The data for Subject 2 & 3 also reflect outliers as depicted in Table 1. Therefore, the data distribution for all the subjects is abrupt in nature which indicates noise in it.

## Dataset pre-processing

**Fig. 6:**
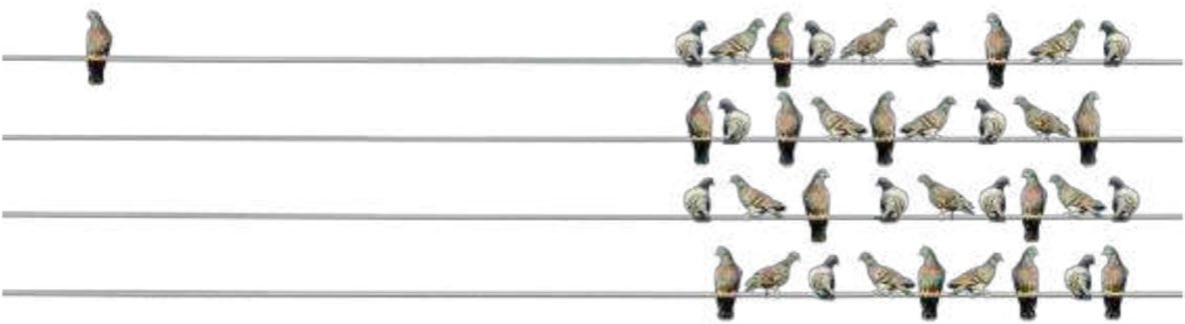
Unusual data point (Outlier) in dataset

Data pre-processing transforms the data into new dimensions by dropping and manipulation. Outliers’ removal is the part of pre-processing which are unusual data point in the dataset as shown in Fig. 6. Usually, their removal helps to get better accuracy for a given dataset. It has been observed that BCI competition 4 dataset IV has outliers as explained in Sect. 2.

**Fig. 7:**
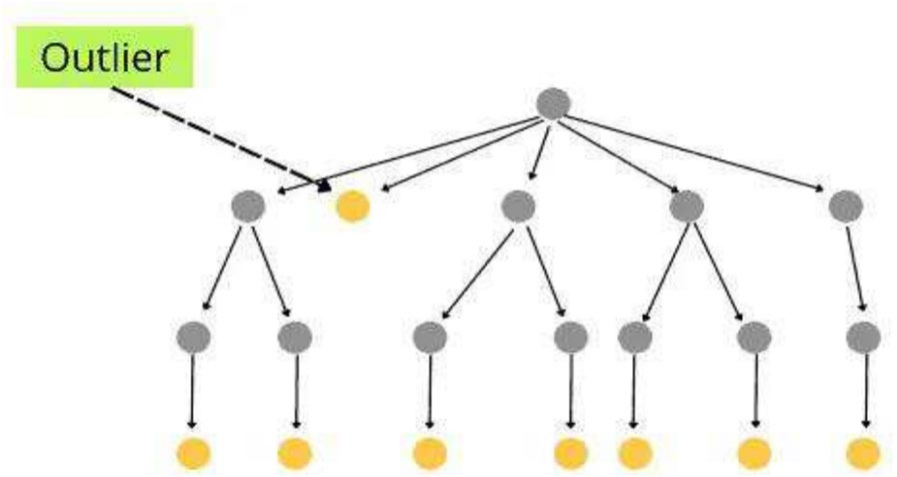
Isolation forest tree

Isolation Forest (IF), Density-Based Spatial Clustering of Applications with Noise (DBSCAN) and Local Outlier Factor (LOF) are prominent outlier removal algorithms. IF stands best among others due to its quantitative isolation and ensemble strength. It works on the concept of tree structure like a random forest algorithm. It takes a random data sample from the dataset as starting and assigns it to a tree. Further, branching is done based on random feature selection. Sub-branching is done based on a random threshold of the feature. Data points are further classified by a tree-like left or right based on the threshold value as shown in Fig. 7.

**Fig. 8:**
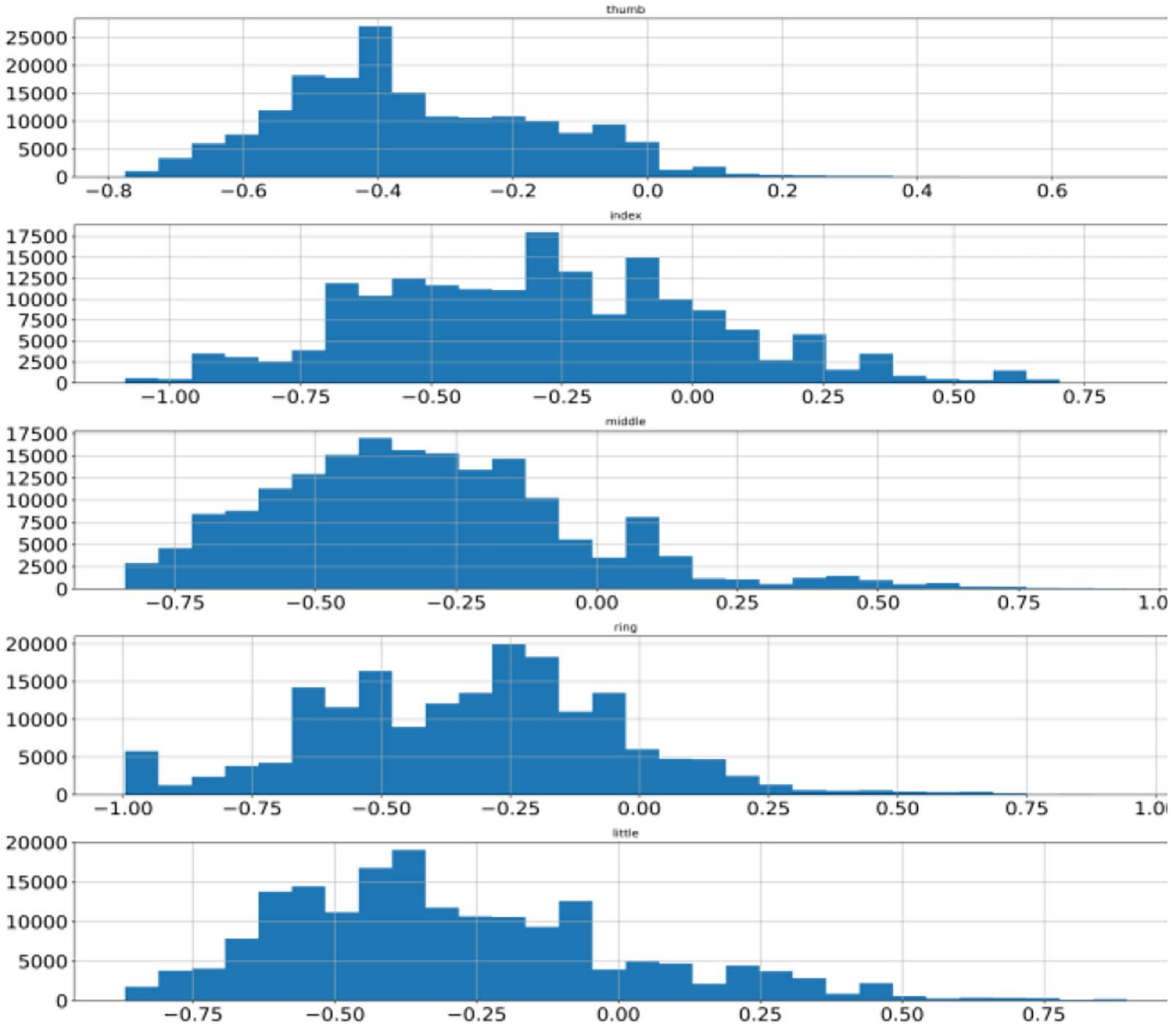
Histogram of subject 1

**Fig. 9:**
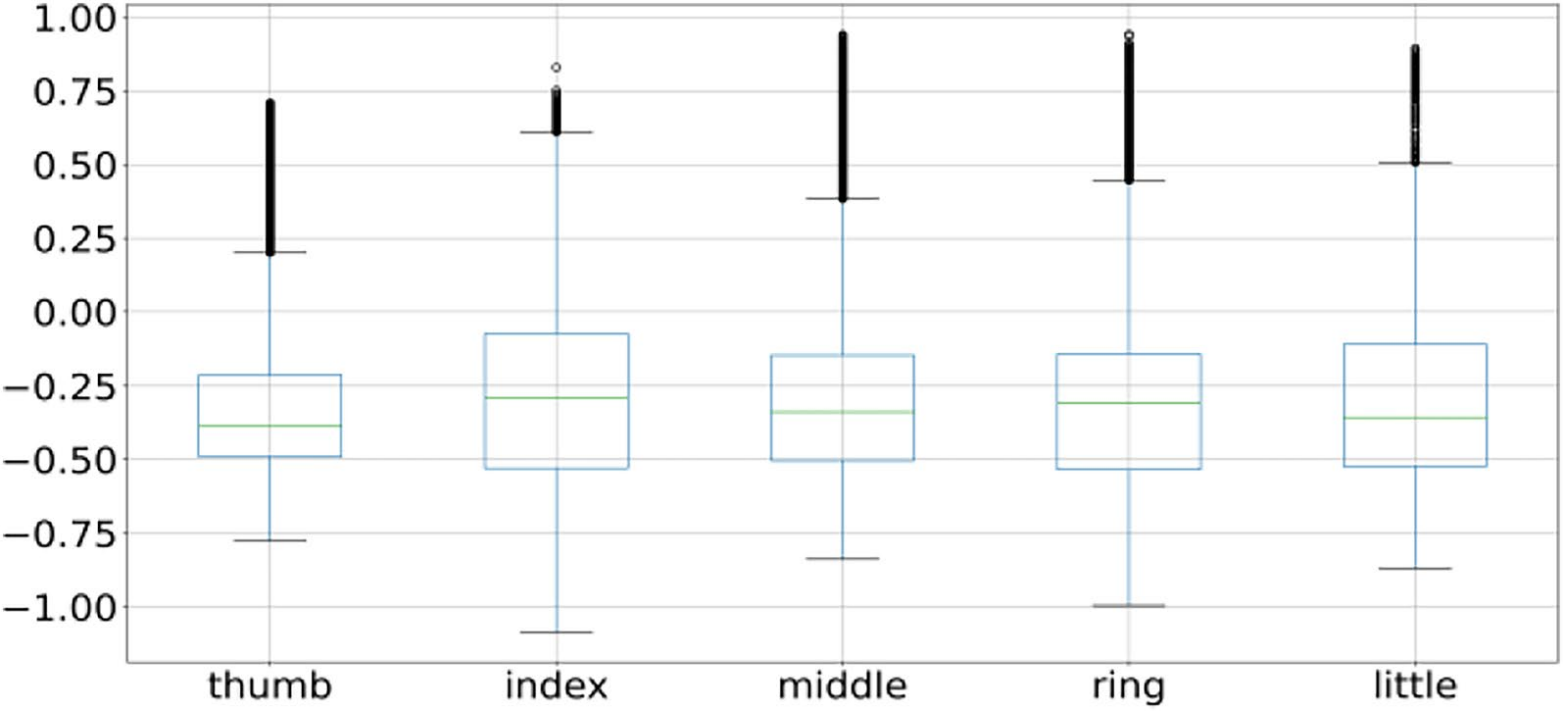
Subject 1 fingers after isolation forest

**Fig. 10:**
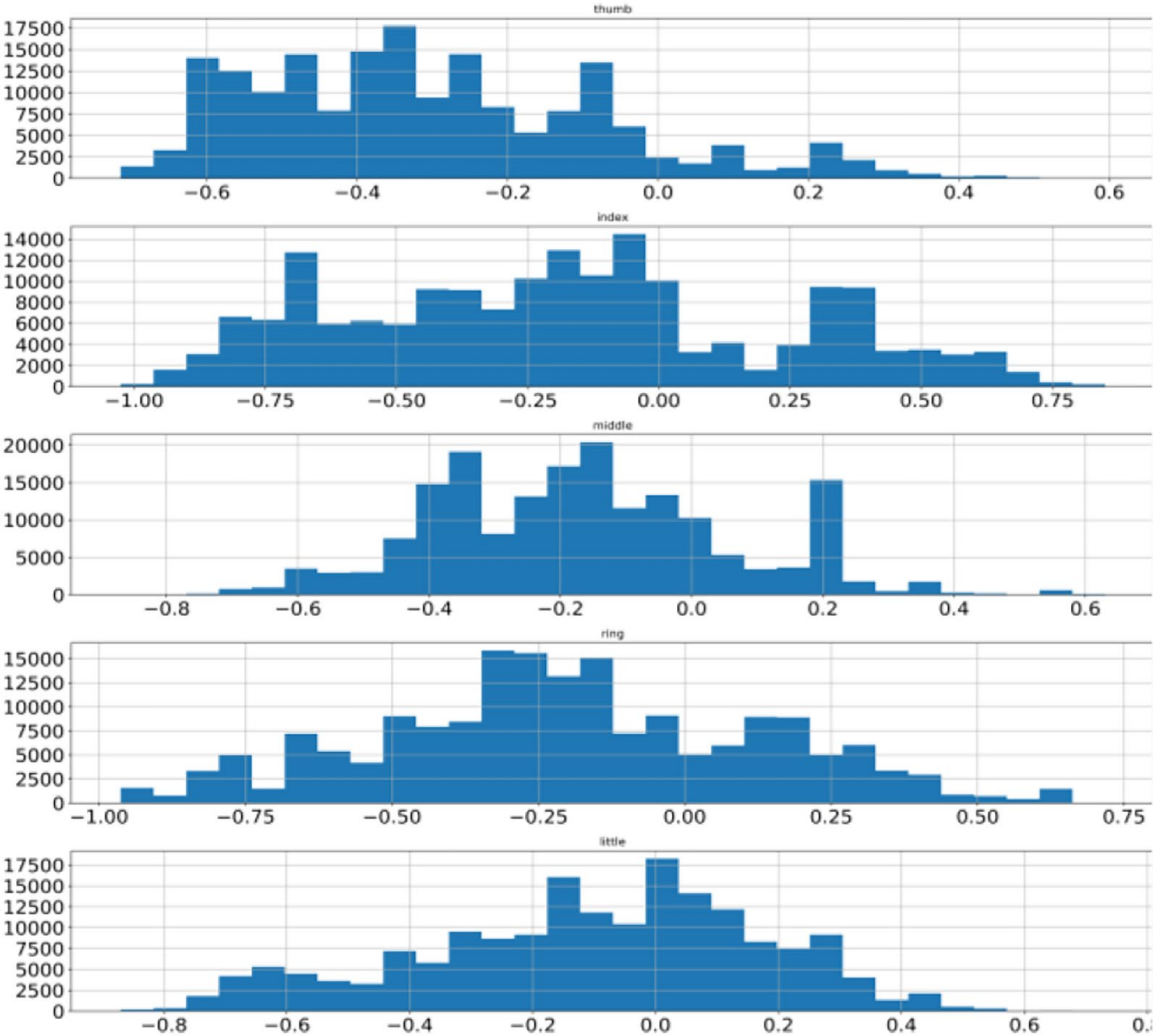
Histogram of subject 2

**Fig. 11:**
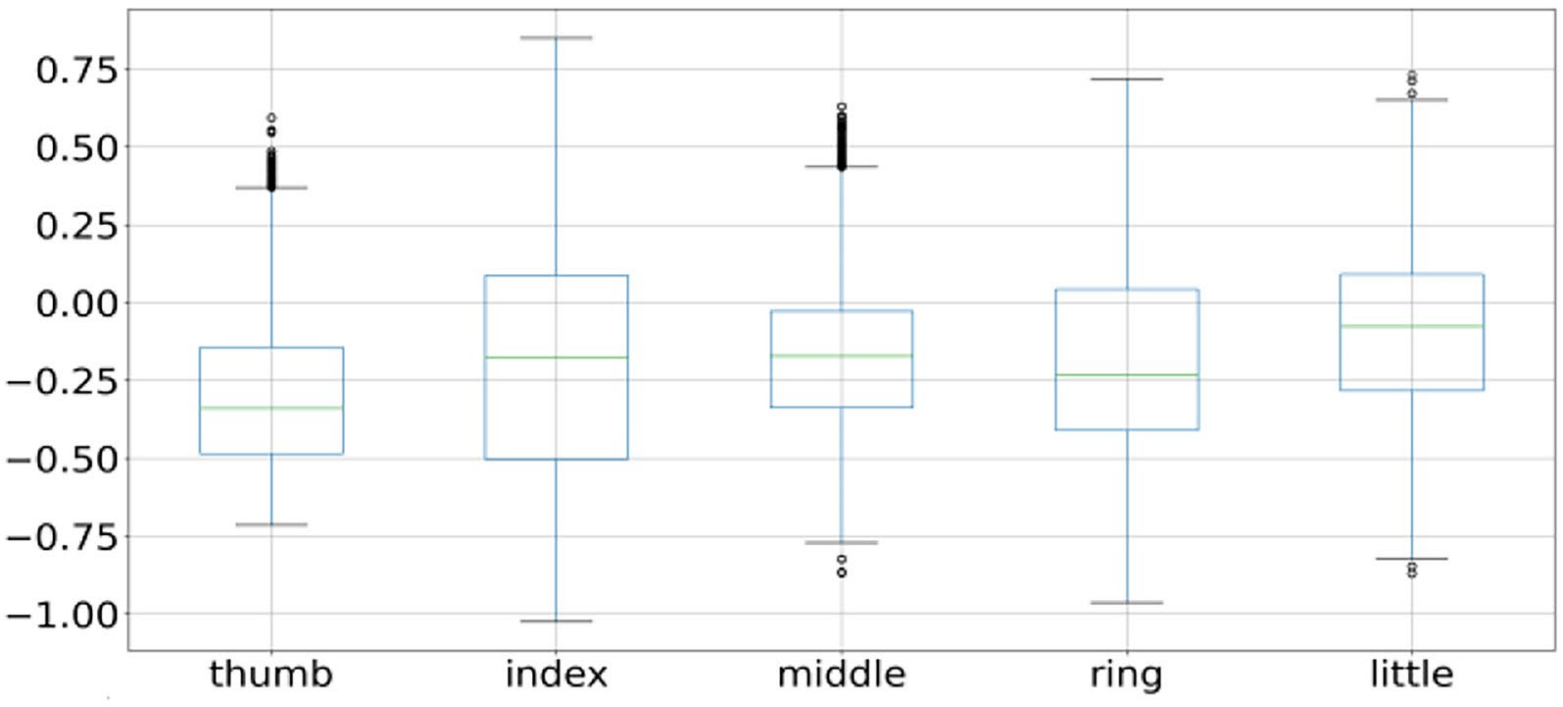
Subject 2 fingers after isolation forest

**Fig. 12:**
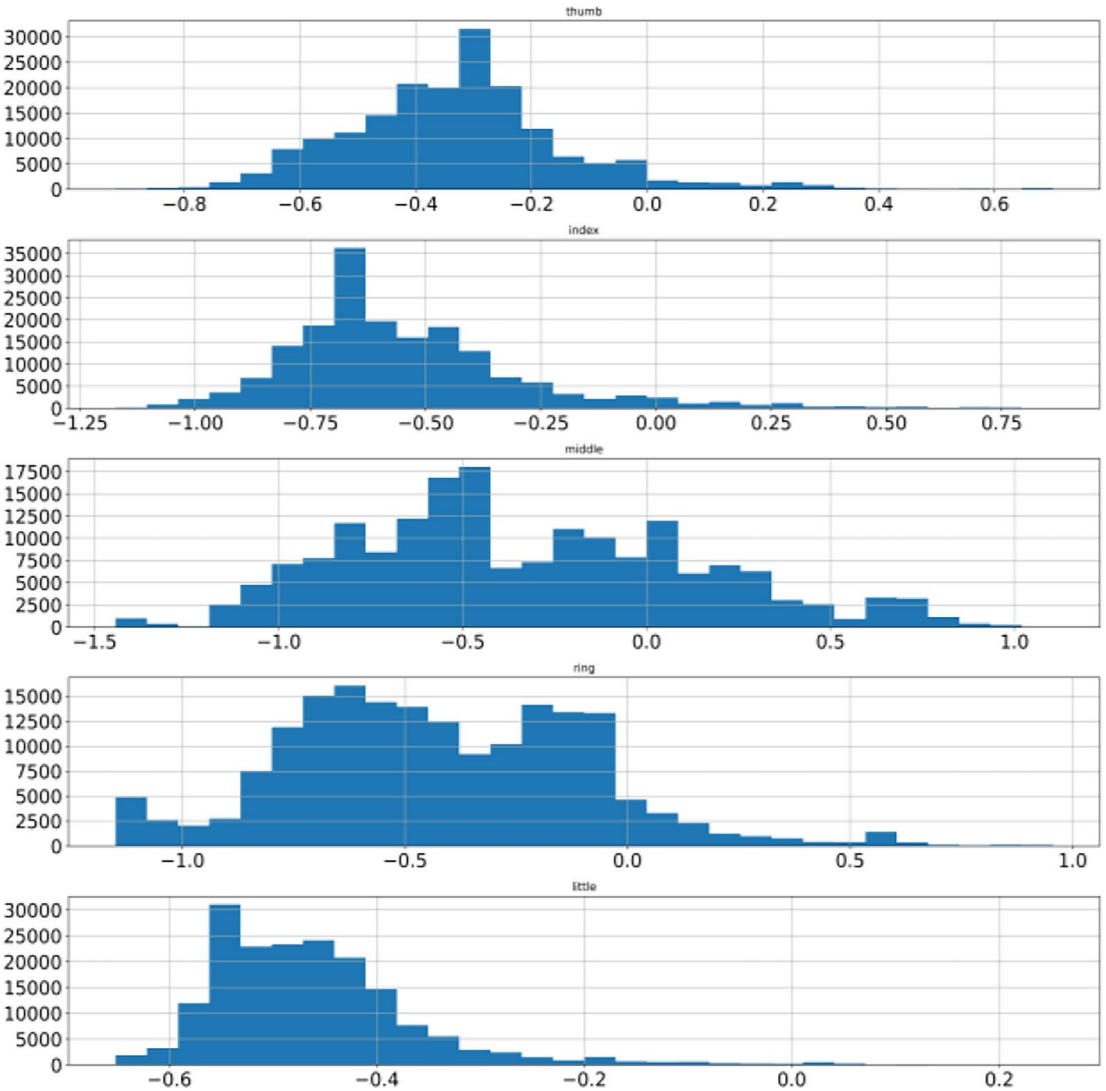
Histogram of subject 3

This process is repeated for the remaining data points. Model training completes when an ensemble of the tree is over. Data points are assigned anomaly scores based on the score from the individual tree and an aggregation score is generated that reflects the level of contamination of that particular data point. In this way, every data point is assigned an aggregated score. A dataset is cleaned by deciding the value of the contamination level. A higher value will remove more data points whereas a low value will remove fewer data points from the dataset. A suitable value needs to be decided using heuristic approach enhancing data quality. Isolation forest is applied on BCI competition 4 dataset iv which transformed data from abrupt nature to near gaussian distribution for all the subjects. Figure 8 shows mean, minimum & maximum value to −0.35, −0.78 and + 0.71 for subject 1 thumb finger. Pre-processing lowered the difference of mean with minimum as well as maximum value as compared to earlier. To visualize the pre-processed dataset histogram is used whereas the differences are shown with box-plot as illustrated in Sect. 2 and shown in Fig. 9 for subject 1. Other two subjects have similar data distribution, as depicted in Fig. 10, 11, 12, 13 showing histogram and boxplot respectively. Table 2 shows post processing effects on the dataset which will help us to infer activation function selection efficiently, reducing epochs and overall improvement in learning rate.

**Fig. 13:**
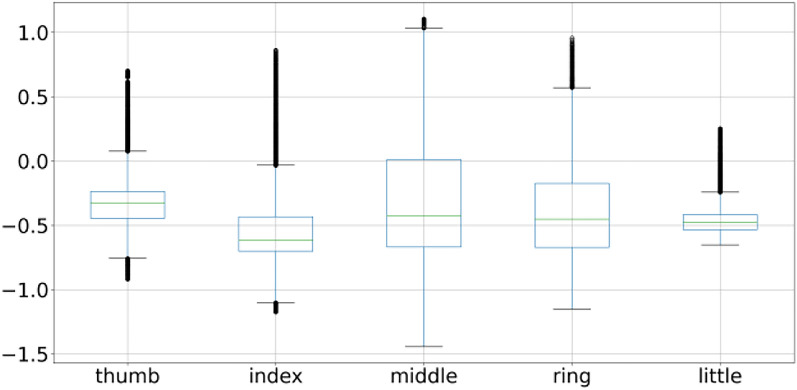
Subject 3 fingers after isolation forest

**Table 2:** Descriptive information of subject 1,2 & 3 after applying the isolation forest

Stats	Finger				
	Thumb	Index	Middle	Ring	Little
Count	18 K	18 K	18 K	18 K	18 K
Subject 1					
Mean	−0.35	−0.29	−0.31	−0.3	−0.29
Std	0.20	0.32	0.28	0.29	0.30
Min	−0.78	−1.09	−0.84	−1.00	−0.87
25%	−0.49	−0.53	−0.50	−0.53	−0.52
50%	−0.38	−0.29	−0.34	−0.31	−0.36
75%	−0.21	−0.07	−0.15	−0.14	−0.11
max	0.71	0.83	0.94	0.94	0.90
Subject 2					
Mean	−0.30	−0.18	−0.16	−0.20	−0.10
Std	0.23	0.41	0.22	0.32	0.28
Min	−0.71	−1.03	−0.87	−0.96	−0.87
25%	−0.49	−0.50	−0.34	−0.41	−0.28
50%	−0.34	−0.17	−0.17	−0.23	−0.08
75%	−0.14	0.09	−0.03	0.04	0.09
max	0.59	0.85	0.63	0.72	0.73
Subject 3					
Mean	−0.33	−0.55	−0.33	−0.42	−0.46
Std	0.20	0.26	0.47	0.33	0.10
Min	−0.92	−1.17	−1.44	−1.15	−0.65
25%	−0.44	−0.70	−0.67	−0.67	−0.53
50%	−0.32	−0.61	−0.43	−0.45	−0.48
75%	−0.24	−0.43	0.01	−0.17	−0.42
max	0.70	0.86	1.10	0.96	0.25

## BC4D4 model

**Fig. 14:**
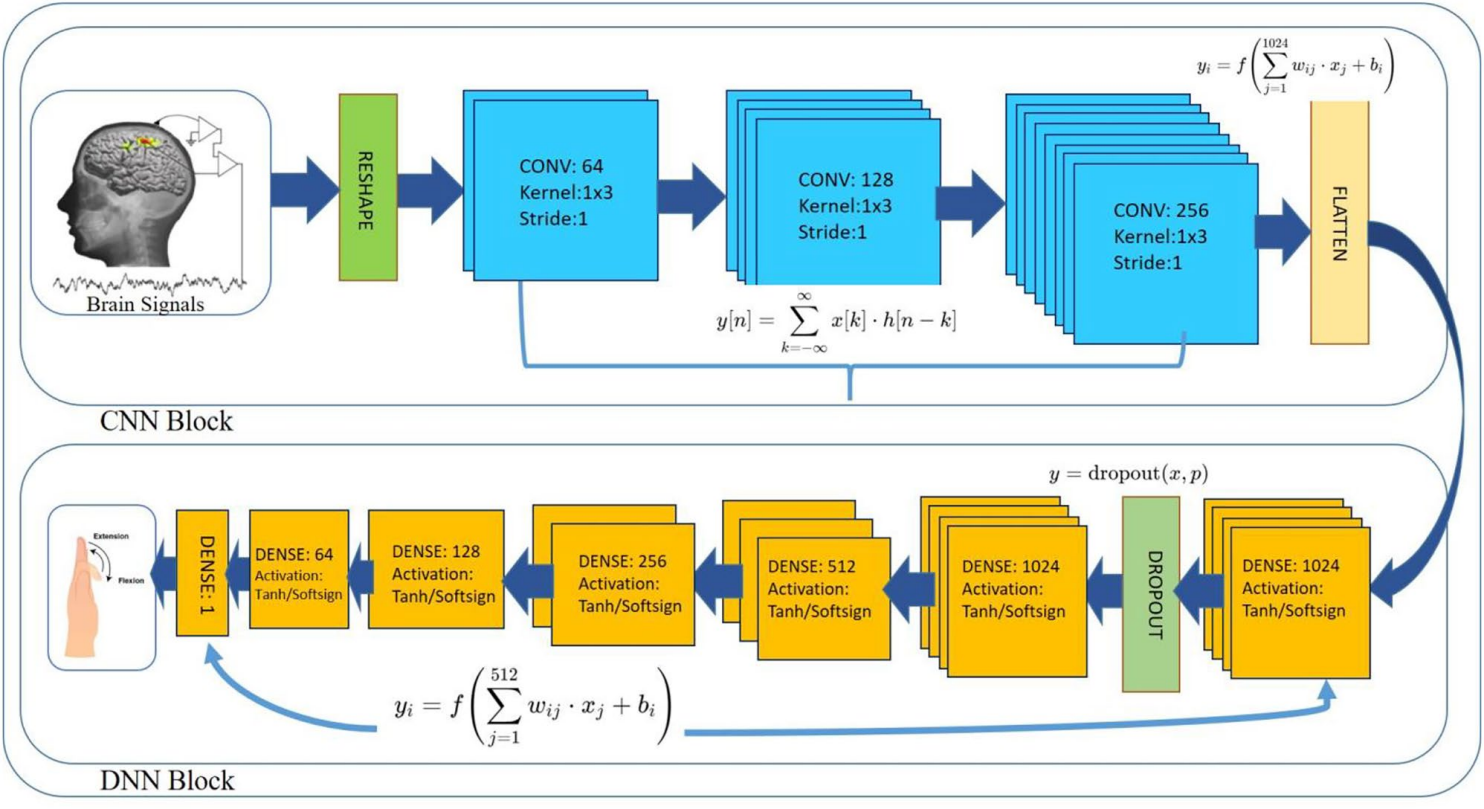
BC4D4 model architecture

The BCI competition 4 Dataset 4 is popular among researcher which inspired us to name our proposed model as BC4D4. Figure 14 shows the BC4D4 model which uses CNN block for feature extraction [31, 35]. CNNs are exceptionally powerful in feature extraction, especially for data with a spatial or temporal structure, such as time series and dataset “BCI Competition 4 dataset iv” contain both spatial and temporal data. CNNs are designed to automatically and adaptively learn spatial hierarchies of features from the data. Early convolutional layers capture low-level features (vertical scaling such as 64, 128, 256), while deeper layers’ capture high-level features (horizontal scaling such as three Dense CNN layers). Pooling layer reduce the dimension of data and due to that loss of information occur and that increases the difficulty for the NN model to infer from small amount of corpus, so pooling layer is not used even-though it works well for the dimensionality reduction. One dimensional kernel is used of size 3, so shape of kernel is 1 × 3 with no padding. Small size of kernel performs well to extract temporal patterns. DNN block incorporate first layer as DNN, second layer as dropout and followed by 5 consecutive DNN layer in size decrement order from 1024 to 64 and last DNN layer of size 1. DNN block fulfill the purpose for pattern identification from the data received from the CNN block. Table 3 list all the parameters of BC4D4 model layer wise. All previous methods stand on several important ideas and improvements over one another. Each suggested approach was unique and have its advantages but overlooked the range of finger movement and its role to find an appropriate activation function defying them to cross the mean accuracy beyond 0.70. The method developed in this study stands on the crucial choice of activation function by inferring typical range of oscillatory activity on the ECoG channels i.e. finger movements and the nature of data distribution. It is observed from Sect. 3 that finger movements lies in both positive and negative range between −1 and + 1. Here IF is used to clean the data following almost gaussian distribution. As we know the activation function like Tanh and softsign covers both positive and negative regions as shown in Fig. 16 allude us to incorporate them in our study before selecting activation function for our model. The present study is first in all with inclusion of Tanh and Soft-sign as activation functions for BCI competition IV dataset 4 and showed a significant improvement compared to all existing solutions.

**Table 3:** BC4D4 tabular parameters

Layer	Type	Input shape	Kernel/weight	Output shape	Param #
0	Conv1D + ReLU	$({X}_{\text{train}}.shape[1],1)$	3 × 1 × 64	$({X}_{\text{train}}.shape[1],64)$	(3⋅1⋅64) + 64 = 256
1	Conv1D + ReLU	$({X}_{\text{train}}.shape[1],64)$	3 × 64 × 128	$({X}_{\text{train}}.shape[1],128)$	(3⋅64⋅128) + 128 = 24,704
2	Conv1D + ReLU	$({X}_{\text{train}}.shape[1],128)$	3 × 128 × 256	$({X}_{\text{train}}.shape[1],256)$	(3⋅128⋅256) + 256 = 98,560
3	Flatten	$({X}_{\text{train}}.shape[1],256)$	–	$({X}_{\text{train}}.shape\left[1\right].256)$	0
4	Dense + Tanh	Flatten Output	256⋅1024	1024	(256⋅1024) + 1024 = 262,144
5	Dropout (0.1)	1024	–	1024	0
6	Dense + Tanh	1024	1024.512	512	(1024⋅512) + 512 = 524,800
7	Dense + Tanh	512	512⋅256	256	(512⋅256) + 256 = 131,328
8	Dense + Tanh	256	256⋅128	128	(256⋅128) + 128 = 32,896
9	Dense + Tanh	128	128⋅64	64	(128⋅64) + 64 = 8,256
10	Dense + Tanh	64	64⋅1	1	(64⋅1) + 1 = 65

BC4D4 model takes subject’s feature as input and returns a regression value as output to predict a particular finger movement. BC4D4 layered architecture has no other changes except the number of features. Subject 1 has 62, subject 2 has 48, and subject 3 has 64 features that will change layered architecture accordingly. The size of input window is a variable that is shown as “ip” in Fig. 16. The mathematical expression for this model is summarized as (1).

1 $$y = \tanh \,\left( {w_{5} \, \cdot \,\tanh \left( {w_{4} \cdot \tanh \,\left( {w_{3} \cdot dropout\,\left( {w_{2} \cdot \tanh \,\left( {w_{1} \cdot flatten\left( {conv3\left( {conv2\left( {conv1\,\left( x \right)} \right)} \right)} \right)} \right)} \right)} \right)} \right)} \right)$$

The features are first reshaped and then passes through a series of convolution layer defined as (2). Here y[m] represents the value at index m in the output feature map, b is the bias term, x[n] represents the value at index n in the input signal, h[m–n] represents the value at index (m—n) in the filter/kernel.

2 $${\text{y}}\left[ {\text{m}} \right] = {\text{ b }} + \,\sum {\left( {{\text{x}}\left[ {\text{n}} \right]{\text{* h}}\left[ {{\text{m}} - {\text{n}}} \right]} \right)} { }$$

Third convolution layer passes the data to the flatten layer that goes further to the dense layer. Dropout layer is sandwiched between two dense layers each having 1024 perceptron. The dense layer is further passing the vector to 512-perceptron layer and continues to 256 perceptron, 64 perceptron and lasts to single perceptron in final layer. Our proposed architecture uses tanh or softsign as activation function with all the dense layers and results in two different models having the same layered architecture except their activation functions.

Activation functions play a crucial role when working with a dataset like BCI 4 dataset that is highly distributed (positive and negative) in nature and every subject response is varied even though visual cues are the same. Individual finger movement range between −1.0 and 5, but the maximum movement range lies in −1 to + 1 as shown in Sect. 2 box plots. Sigmoid is the most common activation function used in a neural network, where the range of datasets vary from 0 to + 1 whereas Rectified Linear Unit eLU) is preferred when the range starts from 0 to positive region. Mathematically these can be shown as (3), (4).

3 $${\text{Sigmoid}}\quad f\left( x \right) = \frac{1}{{1 + e^{ - x} }}$$

4 $${\text{Relu}}\quad f\left( x \right) = \left\{ {\begin{array}{*{20}c} 0 & {{\text{ for }}x < 0} \\ x & {{\text{ for }}x \ge 0} \\ \end{array} } \right.$$

**Fig. 15:**
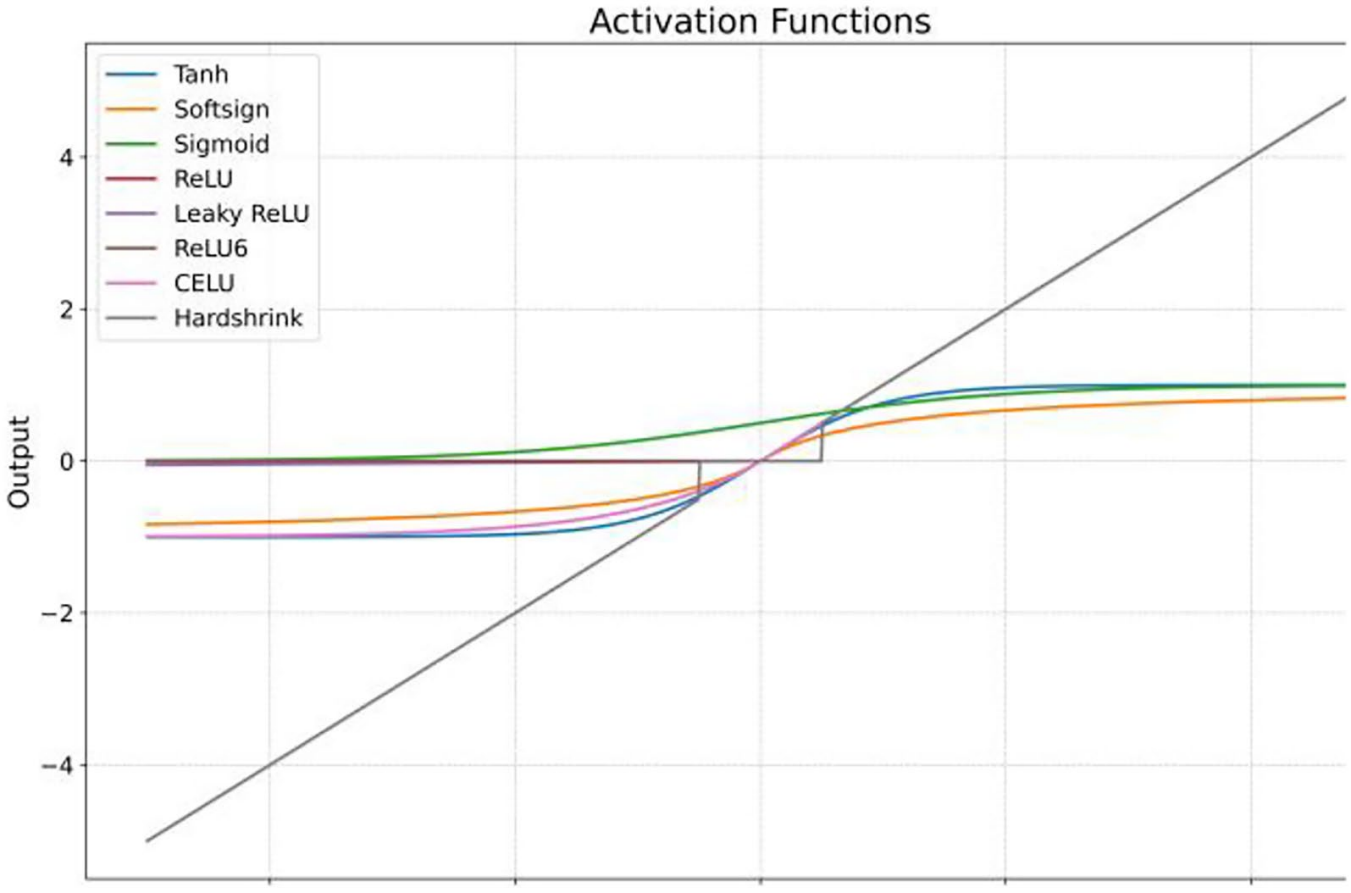
Activation functions

On contrary to (3) & (4) Leaky Rectified Linear Unit (LeakyReLU) covers both positive and negative region as in (5). It is linear in both regions which makes it unsuitable for our model. Tanh and softsign are nonlinear in both positive and negative regions make them suitable activation function for our proposed model as shown in Fig. 15. Tanh and softsign can be represented as (6) and (7).

5 $${\text{LeakyRelu}}\quad f\left( x \right) = 0.01x$$

6 $${\text{Tanh}}\quad f\left( x \right) = \frac{2}{{1 + e^{ - 2x} }} - 1$$

7 $${\text{Soft Sign}}\quad f\left( x \right) = \frac{x}{{\left( {1 + \left| x \right|} \right)}}$$

8 $${\text{Relu 6}}\quad f\left( x \right) = min\left( {max\left( {0,x} \right),6} \right)$$

9 $${\text{Celu}}\quad f\left( x \right) = \,\left\{ \begin{gathered} x\quad \quad \quad \quad \quad \quad \quad \quad \,ifx> 0, \hfill \\ \alpha \cdot \left( {{\text{exp}}\left( {\frac{x}{\alpha }} \right) - 1} \right)\quad \quad ifx \le 0. \hfill \\ \end{gathered} \right.$$

10 $${\text{HardShrink}}\quad f\left( x \right) = \,\left\{ \begin{gathered} x\quad \quad if\left| x \right|> \lambda , \hfill \\ 0\quad \quad if\left| x \right| \le \lambda . \hfill \\ \end{gathered} \right.$$

**Fig. 16:**
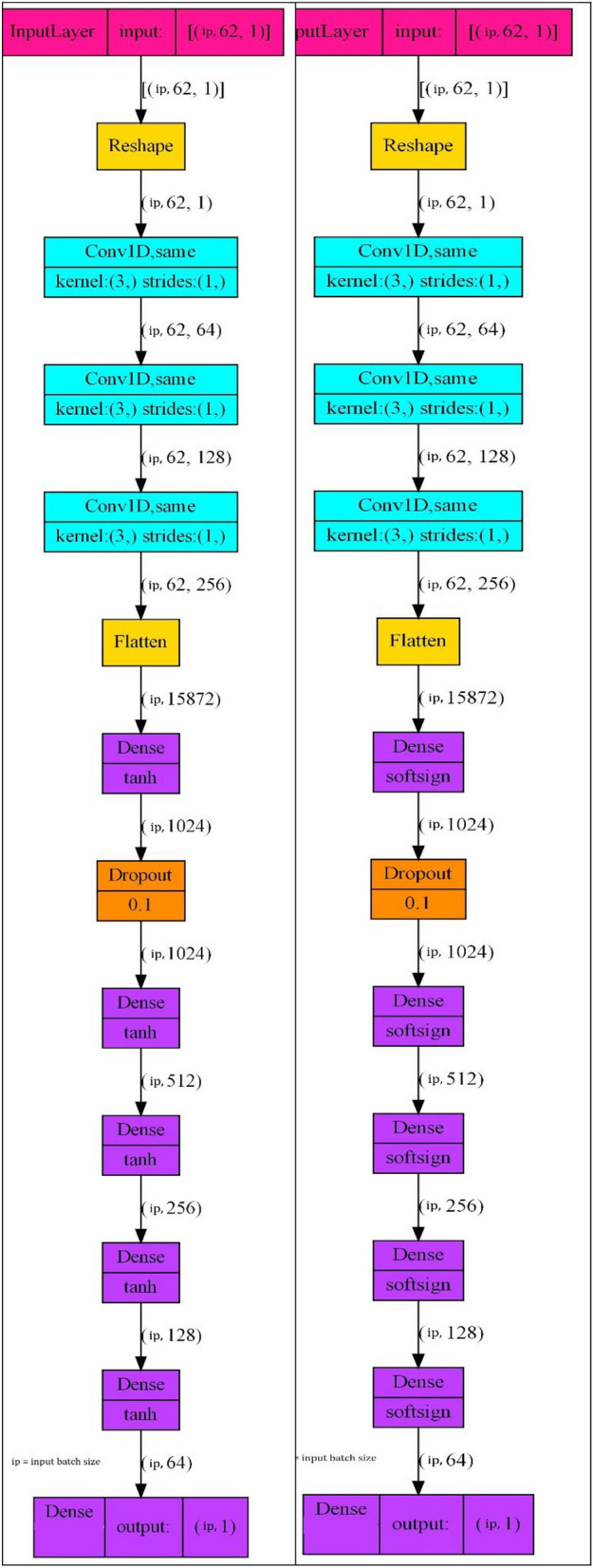
BC4D4 model layered architecture

The tanh activation function is an ‘S-shaped curve and play important role especially in regression category where the dataset value lies between −1 and + 1 as in case of BCI dataset. ReLU6 is the limited version of Relu and limit is 6 in positive region (Eq. 8). Continuously differentiable exponential linear (Celu) is suitable for linear data that lies in the range of −1 to positive region (Eq. 9). Hardshrink shrinks values near to zero to all the input in the ragion of it and for other values its behavior is linear (Eq. 10) as shown in Fig. 15. ReLU and its variants (ReLU6, CELU) suffer from the "dying ReLU" problem where neurons can become inactive if their inputs are consistently negative, leading to zero gradients. While CELU mitigates this to some extent with its negative part, it can still be less effective than tanh and softsign, if the data distribution heavily favors negative inputs and this dataset favors values in both regions. Figure 16 shows the layered architecture of BC4D4 with tanh and softsign activation function. Our model correlation performance for all the subjects with tanh activation is shown in Table 4. Our model result followed a pattern in terms of average accuracy of subjects which reveals that the average accuracy of subject 3 and subject 1 have higher accuracy ~ 85% comparing to subject 2 which is ~ 70%. Further, individually thumb has shown higher average accuracy among rest of the finger set of any user whereas the ring finger has achieved highest correlation value i.e. 93.

**Table 4:** BC4D4 Tanh model performance on individual finger

Sub. No	Thumb	Index	Middle	Ring	Little	Avg
Sub-1	0.89	0.87	0.85	0.86	0.88	0.87
Sub-2	0.79	0.66	0.45	0.70	0.78	0.68
Sub-3	0.89	0.86	0.90	0.93	0.83	0.88

The correlation performance for all the subjects with softsign is higher as compared to tanh activation is shown in Table 5. The result in Table 5 followed same pattern in terms of average accuracy of subjects which reveals that the average accuracy of subject 3 and subject 1 have higher accuracy ~ 90% comparing to subject 2 which is ~ 80%. Softsign worked more effectively on subject 2 improving lowest correlation value by ~ 44% for middle finger. Individually ring finger has shown higher average accuracy as well as highest correlation value i.e. 93.

**Table 5:** BC4D4 Softsign model performance on individual finger

Sub. No	Thumb	Index	Middle	Ring	Little	Avg
Sub-1	0.88	0.86	0.84	0.86	0.86	0.86
Sub-2	0.82	0.81	0.79	0.83	0.80	0.81
Sub-3	0.91	0.85	0.91	0.93	0.90	0.90

**Fig. 17:**
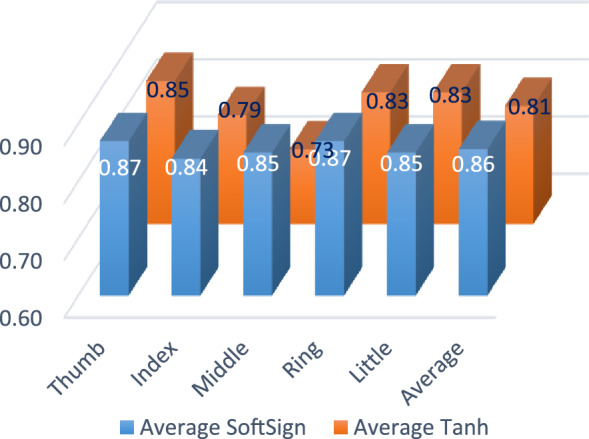
Correlation value of BC4D4 with softsign & tanh

It is observed from the tables clearly that Softsign has outperformed over tanh activation function. Softsign mapped the dataset values because this function grows poly-nominally rather than exponentially as in case of tanh function. Moreover, the gentler non-linearity of softsign results in better and faster learning as it never struggles with vanishing gradient problem. This improves the average correlation values of the individual finger of any subject for the BCI dataset as compared to tanh function as shown in Fig. 17. Here the average correlation of BC4D4 with softsign activation function is 5% more than the BC4D4 with tanh activation function without altering the hyper parameters of the model.

Table 6 represents a state of art comparative study of models implemented on BCI dataset. The dataset gain popularity in 2012 with Flamary standing 2nd place in the competition attaining a 0.43 average correlation value. His model used switching linear model with auto regressive as an activation function but the model of Liang and Bourgain claimed 0.48 correlation value and begged the winner position of the competition [59, 60]. Their model used simple linear regression with band specific feature engineering. Xie et al. used LSTM with embedded sigmoid and tanh activation function crossing correlation value upto 0.52 [59, 60]. In year 2021 first Frey et al. used multi-purpose CNN and later Petrosyan et al. with interpretable CNN achieving the overall average performance as 0.52 and 0.45 values respectively [59, 60]. Both their model used Relu as activation function but interpretable CNN lacked due to feature engineering. Recently Yao et al. used lightGBM increasing the overall average near to 0.53 value. Their model used Riemannian features which is frequently used in many BCI applications and implementations. The fingerFlex model by Lomtev et al. is first which crossed the correlation values upto 0.67 in the year 2022 [59, 60]. Their model used CNN for the first time with a new activation Gaussian Error Linear Unit (GELU).

**Table 6:** State of art model implemented on BCI dataset

Author	Model	Activation F(x)	Subject wise (Avg)		
			I	II	III
Flamary (2012)	Switching Linear	Auto-regressive	0.48	0.24	0.56
Liang et al. (2012)	Linear Regression	Feature engineering	0.45	0.39	0.59
Xie et al. (2018)	LSTM	Sigmoid & Tanh	0.56	0.41	0.58
Frey et al. (2021)	Multi-purpose CNN	Relu	0.52 (avg)		
Petrosyan et al. (2021)	Interpretable CNN	Relu	0.45	0.34	0.56
Yao et al. (2022)	Light GBM	Riemannian features	0.52	0.47	0.61
FingerFlex (2022)	CNN	GELU	0.66	0.62	0.74
Proposed BC4D4	CNN + DNN	IF with Tanh	0.87	0.67	0.88
		IF with Softsign	0.86	0.81	0.90

Proposed model BC4D4 model has worked on data preprocessing to remove the outlier and later introspecting the dataset and its trend. Further inspecting the finger movement range for a subject i.e. (+ 1 to −1) made BC4D4 to choose tanh and Softsign to use the activation function which led the performance crossing average correlation value above 0.81 and 0.85 respectively. This is maximum accuracy claimed with till date on the BCI competition 4 dataset iv for the finger movement. Further it removes the usages of different filters which creates delay in real time processing like finite impulse response, band-pass filter, or band stop filter to remove noise or outlier removal. The model removed training phases as needed to classify rest or moving finger states, further it uses IF which removes outliers making it faster and suitable for real time implementation. BC4D4 use of neural network in model enhances its flexibility to extracts patterns making its window length variable to perform more realistic.

**Fig. 18:**
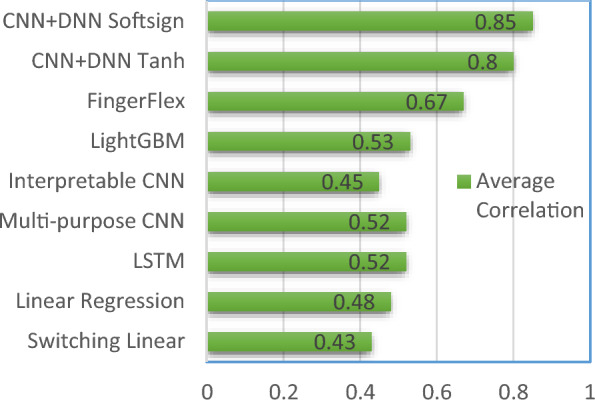
Models comparison

It is always challenging for a model to extract the features specially when the electrode placement is not identified and marked in the dataset [61]. This usually affects the feature engineering which is a prominent step in model design. BC4D4 attempted in a defined range of finger movements (+ 1 to −1) limits its generalization however it outperformed and reached to highest correlation value as compared to all the existing model which used BCI dataset till date and known to us. The model comparison is presented in Fig. 18.

## Conclusion

The paper presented use of conventional statistical methods to enhance visualization which helped in extraction of dataset nature, here BCI4. It also suggested isolation forest as an outlier removal algorithm improving overall quality of data. Further the paper emphasized the step to choose activation function carefully before modelling aiming higher accuracy. A new model is proposed using CNN and DNN i.e. BC4D4 which improved 1.85 times accuracy than winner models of BCI 4 competition and 1.25 times than most recent FingerFlex model as well. In nutshell it outperformed over all existing state-of-the-art approaches claiming highest correlation value 0.85 with softsign and tanh activation function. This work will be a promising step to use deep learning techniques for BCI applications.

## Data Availability

No datasets were generated or analysed during the current study.
